# Chromosomal Abnormalities in *Allium cepa* Induced by Treated Textile Effluents: Spatial and Temporal Variations

**DOI:** 10.1155/2020/8814196

**Published:** 2020-08-03

**Authors:** W. M. Dimuthu Nilmini Wijeyaratne, P. G. Minola Udayangani Wickramasinghe

**Affiliations:** Department of Zoology and Environment Management, Faculty of Science, University of Kelaniya, Kelaniya, Sri Lanka

## Abstract

Appropriate effluent treatment processes are expected to significantly reduce the toxicity of effluents before they are released to the natural environment. The present study was aimed to assess the spatial and temporal variations of the physical and chemical water quality parameters of a natural water body receiving treated textile effluents and to assess the chromosomal abnormalities induced by the treated textile effluents. Four sampling sites (A: effluent discharge point; B: 100 m downstream from site A along the tributary; C: 200 m downstream from site A along the tributary; D: 100 m upstream from site A along the tributary) were selected associated to a tributary that received treated textile effluent. The physical and chemical water quality parameters were measured in the composite water samples collected from the study sites, and *Allium cepa* bioassay was conducted using aged tap water as the control. Sampling was conducted in both rainy and dry seasons. The conductivity, TDS, COD, and colour intensity of the water samples collected from the study sites were significantly higher during the dry season compared to those in the rainy season. *Allium cepa* root meristematic cells exposed to water samples from sites A, B, and C showed a significantly high interphase and prophase indices compared to those exposed to aged tap water and upstream site during both rainy and dry seasons. The mitotic index of the root tip cells of *Allium cepa* bulbs exposed to the water samples collected from the effluent discharge point (site A) and from the 100 m downstream site from site A (site B) was significantly lower than that of the other sites in both rainy and dry seasons. However, the mitotic index of the root tip cells of *Allium cepa* bulbs exposed to the water samples from the upstream site was not significantly different from that of the control treatment during both sampling seasons. The bioassay indicated that the mitotic index and phase index of the root meristematic cells of *Allium cepa* can be affected by the treated textile effluents released to the water body and the occurrence of C metaphase, chromosomal adherence, bridges, disturbed anaphase, vagrant chromosomes, and chromosomal breaks indicated that the treated textile effluent receiving tributary can possibly contain genotoxic and mutagenic compounds which can induce chromosomal abnormalities.

## 1. Introduction

Textile industry is a highly water consuming industry and produces large amount of wastewater [1]. Release of textile industry wastewater into the natural aquatic environment can induce environmental pollution and result in health risks to humans and other organisms. Therefore, treatment of textile wastewater to remove the high levels of suspended solids, dyes, salts, nonbiodegradable organic compounds and heavy metals is identified as a sustainable option to reduce pollution of the natural aquatic systems [2, 3]. The physical and chemical parameters of the treated textile effluents are monitored before releasing them to the natural environment to assure that they comply with the textile effluent discharge standards [4]. However, once these treated effluents are released to the natural environment, their effects on the feral organisms are rarely monitored and recorded. Some compounds including organic chemicals and heavy metals even at very low concentrations can impose chronic and acute effects on the organisms living in the natural environment [5–8]. Therefore, it is very important to monitor the environmental effects and possible health effects imposed on the feral organisms by the released treated effluents. This monitoring can be conducted by measuring the physical and chemical water quality parameters of the released environment over a certain period of time or along a spatial gradient from the discharge point [5, 8]. In addition to the water quality parameters, the measurements on biological parameters can also provide valuable information about the effects of these released treated effluents on the biological and ecological quality of the ecosystem [7, 9]. For the purpose of measuring biological parameters, the diversity or abundance related studies and in situ or ex situ biological assays can be conducted.

Among the biological assays, plant based bioassays are widely practiced by many researchers [8, 9]. These plant based bioassays are rapid, inexpensive, do not require elaborate laboratory facilities, and have a wide range of genetic endpoints. Most plant species are very sensitive to slight changes in the water quality and the responses are easily detectable as early warning signs. In addition, there are several advantages of using plant bioassays over microbial and mammalian systems in assessing cytotoxicity and genotoxicity. Due to the similarity in the chromosomal morphology of plants and mammals, both groups show similar responses to mutagens. Among the higher plants species used for cytotoxicity and genotoxicity testing, *Allium cepa* (common onion) has been recognized as an ideal phytoindicator to assess DNA damages, such as chromosomal aberrations and disturbances in the mitotic cycle. *Allium cepa* bioassay is widely used in cytotoxic and genotoxic studies due to the presence of good chromosomes conditions, such as large chromosomes and in a reduced number (2*n* = 16) [6, 7, 9, 10].

The present study was conducted with the objective of assessing the chromosomal abnormalities in the *Allium cepa* root tip cells exposed to the treated textile effluents released to the natural aquatic environment. This study would provide valuable information about the presence of genotoxic and/or mutagenic substances in the treated textile effluents which can induce genetic abnormalities in the biological organisms in the effluent receiving environment.

## 2. Materials and Methods

### 2.1. Study Area and Sampling Sites

**Figure 1:**
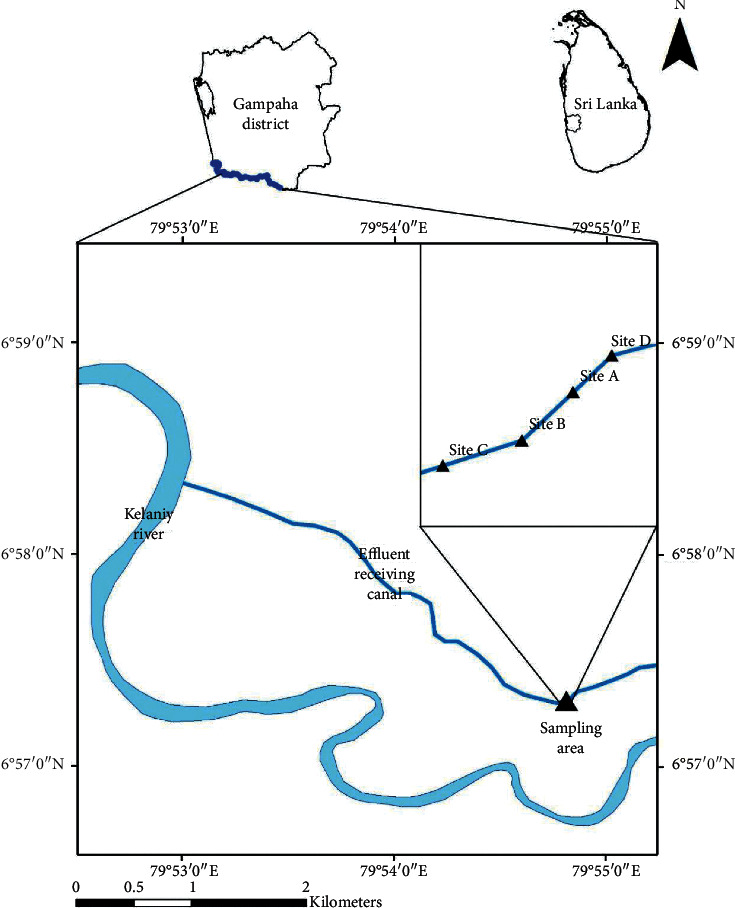
Map of the study area showing the sampling sites. Site A effluent discharge point, site B 100 m downstream from site A, site C 200 m downstream from site A, and site D 100 m upstream from site A.

Sampling sites were selected along a tributary which receives several point source inputs of wastewater treatment plants. The present study focused on a treated textile effluent receiving inlet and four sampling sites were selected covering the input point and upstream and downstream locations. The location of sampling sites is given in Figure 1. Site A was the effluent discharge point, site B was located 100 m downstream from site A, site C was located 200 m downstream from site A along the tributary. Site D (reference site) was located 100 m upstream from site A along the tributary (Figure 1).

### 2.2. Water Quality Parameters

Sample collection and analyses of water quality parameters followed the procedure described in Wijeyaratne and Wickramasinghe [11]. Surface water samples were collected from each site for water quality analysis and toxicity analysis. Sampling was conducted during the operation period of the textile industry in both the dry (June to August) and rainy seasons (September–November) in 2018. In each sampling event, time integrated composite samples were taken from each site to represent a particular subsample at 2-hour intervals during the sampling period from 10:00 h to 14:00 h. The water pH, temperature, conductivity, total dissolved solids (TDS), dissolved oxygen concentration (DO), and salinity were measured in situ using a calibrated digital multi parameter (YSI Environmental Model-556 MPS). In the laboratory, biochemical oxygen demand 5 days after incubation (BOD_5_), chemical oxygen demand (COD), ammoniacal nitrogen and colour of water were analyzed by the following standard methodologies described by the American Public Health Association [12]. Water samples were acidified and were analyzed for Cu and Zn concentrations in the Atomic Absorption Spectrophotometer (Analytic jena (Model NovAA 400p)) [11].

### 2.3. *Allium cepa* Bioassay

The bioassay was conducted by following the procedure as described in Wijeyaratne and Wadasinghe [13]. Commercial variety of common onion (*Allium cepa*) was used for the determination of different parameters of meristematic cells as indicators of cytotoxicity, genotoxicity, and mutagenicity. Equal size healthy onion bulbs were purchased and the loose outer scales were carefully removed and scrapped at the bottom to expose the root primordia. Scraped onion bulbs were germinated in glass test tubes containing distilled water for 24 hours in a dark room. The rooted bulbs were exposed to exposure media (60 mL, composite water samples taken from each site) in glass test tubes. Aged tap water was used as the control medium. 10 onions bulbs were placed on each exposure and control media. Onion bulbs were submerged up to a depth of one-quarter in each test tube. The bioassay was conducted at 25-26°C environmental temperature in a dark room to avoid the direct sunlight. The exposure and control media were renewed daily.

After 48 hours of exposure, ten onion bulbs with growing roots were randomly selected from each exposure media and the control treatment for microscopic studies. Several roots tips (5–8 from each onion bulb) of 1-2 mm length were processed for microscopic studies. Root tips were fixed immediately in ethanol : glacial acetic acid (3 : 1v/v) solution and stored overnight at 4°C. Then root tips were transferred into 70% alcohol and stored at 4°C until analysis. At the time of processing, root tips were placed in hydrochloric acid (1 N) solution for 5 minutes in the incubator at 60°C and washed with distilled water. Root tips were stained with 5% acetocarmine stain for 30 minutes. Then root tips were placed on glass slides with a drop of 5% acetocarmine stain and a cover slip was placed on the glass slide providing a single pressure to squash the tip cells over the slide. Prepared slide for each exposure medium was observed under the light microscope at 400x magnification. Minimum of 1000 *Allium cepa* root meristematic cells were scored from each prepared slide in a random manner to score interphase cells, cells in mitotic stage, and chromosomal aberrations in the dividing cells. The mitotic index (MI) was calculated for root tips of each onion bulb using the following formula (the total number of dividing cells is the cells undergoing prophase, metaphase, anaphase, and telophase stages) [7, 10]:

(1) $$\begin{array}{c} \text{mitotic index}\left(\%\right)=\frac{\text{number of dividing cells counted}}{\text{total number of cells counted}}\times 100. \end{array}$$

The phase indices (PI) were calculated for each onion bulb for interphase and each mitotic stage (prophase, metaphase, anaphase, and telophase) of root meristematic cells using the following formula [7, 10]:

(2) $$\begin{array}{c} \text{phase index (\%)}=\;\frac{\text{number of cells in specific mitotic stage}}{\text{total number of cells counted}}\times 100. \end{array}$$

The % occurrence of each type of chromosomal abnormalities in root meristematic cells was calculated using the following equation [7, 10]:

(3) $$\begin{array}{c} \text{chromosomal abnormalities}\left(\%\right)=\frac{\text{number of cells with specific chromosomal abnormality observed}}{\text{total number of dividing cells }\left(\text{except prophase counted}\right)\;}\times 100. \end{array}$$

### 2.4. Statistical Analysis

After confirming for normality using the Anderson Darling test, the spatial variation of water quality parameters was analyzed using one-way ANOVA followed by Tukey's test. The temporal variation of the water quality parameters during rainy and dry seasons was analyzed using Student's *t*-test. Similarly, the spatial variation of mitotic index, phase index, and chromosomal abnormalities was analyzed using one-way ANOVA followed by Tukey's test and temporal variation was analyzed using Student's *t*-test. Accepted level of significance was *p* < 0.05. MINITAB 14 software was used for statistical analysis of data.

## 3. Results and Discussion

### 3.1. Water Quality Parameters

Spatial variation of water quality parameters during the rainy season is given in Table 1 and during the dry season is given in Table 2.

The temperature, conductivity, TDS, salinity, BOD_5_, COD, ammoniacal nitrogen, Cu concentration, Zn concentration, and the colour intensities at the effluent discharge point were significantly higher than those at the other sites in both rainy and dry seasons (ANOVA, Tukey's test. *p* < 0.05). In both seasons, all these water quality parameters showed a similar pattern of variation among the study sites with the highest concentration at the effluent discharge point (site A) and the lowest concentration in the site 100 m upstream from the effluent discharge point (site D). The variation of the above water quality parameters showed the pattern of site A > site B > site C > site D in both seasons. However, the DO showed a different variation pattern and in both seasons, a significantly high DO was recorded from site D compared to the other sites (Tables 1 and 2, ANOVA, Tukey's test. *p* < 0.05). The DO variation pattern in the study sites followed the pattern of site D > site C∼site B∼site A (Tables 1 and 2) in both rainy and dry seasons.

**Figure 2:**
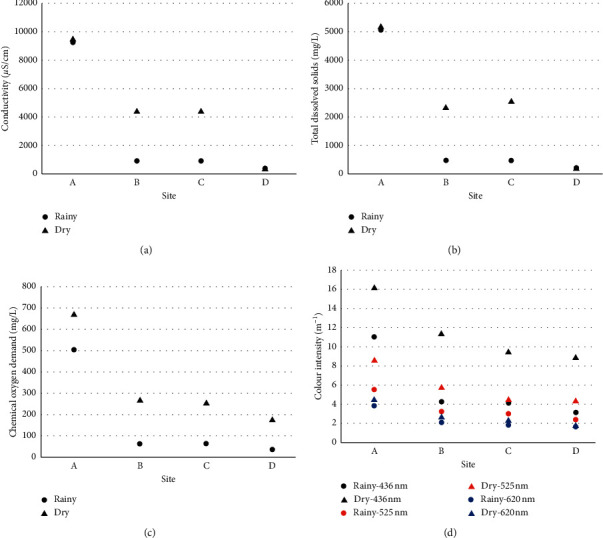
The spatial variation of (a) conductivity, (b) total dissolved solids, (c) chemical oxygen demand, and (d) colour intensity of the water samples collected from the sampling sites during rainy and dry seasons. Parameters during wet and dry seasons are given. Site A effluent discharge point, site B 100 m downstream from site A, site C 200 m downstream from site A, and site D 100 m upstream from site A.

The conductivity, TDS, COD, and colour intensity of the water samples collected from the downstream study sites (sites B and C) during the dry season were significantly higher than that of the rainy season. The spatial variation of these parameters during wet and dry seasons is given in Figure 2. The other water quality parameters did not show significant temporal variation between rainy and dry seasons. The conductivity and TDS at the effluent discharge point (site A) and at the upstream site (site D) in the rainy and dry seasons were not significantly different from each other. However, their concentrations in the two downstream sites (site B: 100 m downstream from the discharge point; site C: 200 m downstream from the discharge point) during the dry season were significantly higher during the dry season than those in the rainy season (Figure 2, Student's *t*-test, *p* < 0.05 COD and colour intensity at 436 nm of the water collected from all the study sites was significantly higher in the dry season than those collected during the rainy season (Figure 2, Student's *t*-test, *p* < 0.05). The dilution effect and the effects of increased flow rate of the tributary during the rainy season may have resulted in low concentrations of these parameters in the study sites. Similar results of the dilution effect on discharged effluent have been reported by Longe and Ogundipe [14] and Islam et al. [15].

### 3.2. *Allium cepa* Bioassay

The mitotic index of the *Allium cepa* root tip cells during the rainy and dry seasons is given in Table 3. The mitotic index of the root tip cells of *Allium cepa* bulbs exposed to the water samples collected from the effluent discharge point (site A) and from the 100 m downstream site from site A (site B) were significantly lower than that of the other sites in both rainy and dry seasons (Table 3, ANOVA, Tukey's test. *p* < 0.05). However, the mitotic index of the root tip cells of *Allium cepa* bulbs exposed to the water samples from the upstream site was not significantly different from that of the control treatment during both sampling seasons (Table 3, ANOVA, Tukey's test. *p* < 0.05). Further, the mitotic index of the root tip cells of *Allium cepa* bulbs exposed to the water samples collected from the effluent discharge point (site A) and from the 100 m downstream site from site A (site B) during the dry season was significantly lower than that of the rainy season (Table 3). The mitotic index in the *Allium cepa* bioassay is considered as an indicator of rhizotoxicity and this can result due to single or a mixture of pollutants present even in very low concentrations [16, 17]. A similar study conducted in Kelani River, Sri Lanka, to assess the cytotoxicity and genotoxicity of treated effluents originated from four types of industrial activities showed that reduction of mitotic index of onion bulbs exposed to the undiluted effluents ranged from 44 to 58% in comparison to the dilution water. They suggested that reduction of mitotic index may be due to the interaction of heavy metals even at trace levels and the presence of many other cytotoxic chemicals in the effluents [18]. Further, a mitotic index less than 22% is considered as a lethal condition for the organisms [13, 19]. In the present study, the mean mitotic index for the samples tested in the rainy season ranged from 26.4% to 52.4%. The mean mitotic index for the samples tested during the dry season ranged from 18.9% to 55.6% and the mean mitotic index at sites A and B was 18.9% and 20.9%, respectively, indicating that the water at those sites during the dry season can cause lethal effects on the organisms (Table 3).

The phase index of the *Allium cepa* root tip cells during the rainy season is given in Table 4 and during the dry season is given in Table 5. During the rainy season, the interphase index of the *Allium cepa* root meristematic cells exposed to water samples from study sites ranged from 580‰ to 790‰. The prophase index of the *Allium cepa* root meristematic cells exposed to water samples from study sites ranged from 278‰ to 440‰. *Allium cepa* root meristematic cells exposed to water samples from sites A, B, and C showed a significantly high interphase and significantly low prophase indices compared to those exposed to aged tap water and upstream site (site D) during the rainy season (ANOVA, Tukey's test, *p* < 0.05, Table 4). These sites showed significantly low telophase index compared to the control group and site D (ANOVA, Tukey's test, *p* < 0.05, Table 4). The interphase, prophase, and telophase indices of the upstream site were not significantly different from that of the control treatment (ANOVA, Tukey's test, *p* < 0.05, Table 4). The anaphase and the metaphase indices of the experimental groups were not significantly different from those of the control group (ANOVA, Tukey's test, *p* < 0.05, Table 4).

During the dry season, the interphase index of the *Allium cepa* root meristematic cells exposed to water samples from study sites ranged from 538‰ to 790‰. The prophase index of the *Allium cepa* root meristematic cells exposed to water samples from study sites ranged from 180‰ to 450‰. Similar to the rainy season, *Allium cepa* root meristematic cells exposed to water samples from sites A, B, and C showed a significantly high interphase and prophase indices compared to those exposed to aged tap water and upstream site (site D) in the dry season as well (ANOVA, Tukey's test, *p* < 0.05, Table 5). Anaphase index and the telophase index of the *Allium cepa* root meristematic cells exposed to water samples from study sites during the dry season were not significantly different from each other nor from the control treatment (Table 5). However, the metaphase index recorded for the effluent discharge site in the dry season was significantly lower than that of the other sites and the control treatment (ANOVA, Tukey's test, *p* < 0.05, Table 5). The metaphase index recorded for the upstream site was not significantly different from that of the control treatment (ANOVA, Tukey's test, *p* > 0.05, Table 5).

Phase index is used to evaluate the inhibition of mitotic cell division. Phase index can be calculated for different phases of cell division. If the phase index of a particular cell division phase is higher, it indicates that the cells in that phase are taking longer time than normal to divide and to enter into next phase [20]. The results of the present study, the interphase, prophase, and telophase indices of the *Allium cepa* bulbs exposed to composite water samples collected from the study sites A, B, and C recorded significantly high interphase and prophase indices and significantly low telophase index compared to the upstream site and control treatment (Tables 4 and 5). Presence of trace metals and other unidentified cytotoxic chemicals in the study sites may have caused these variations in the phase indices which leads to mitosis suppression.

**Figure 3:**
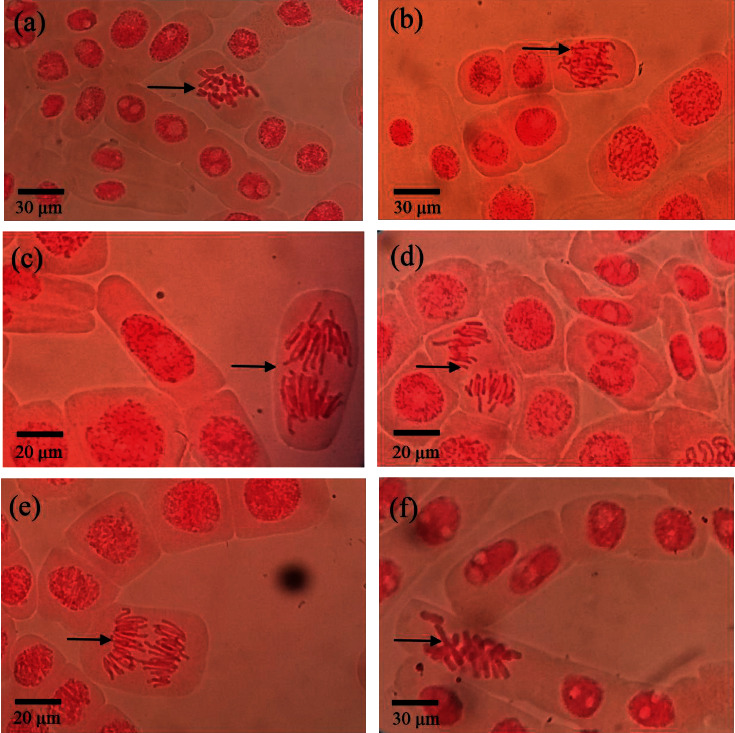
Chromosomal abnormalities observed in *Allium cepa* root meristem following exposure to water samples collected from the study sites. (a) C-metaphase, (b) chromosomal adherence, (c) vagrant, (d) disturbed anaphase, (e) chromosomal bridge, and (f) chromosomal breaks.

The mean chromosomal abnormalities in *Allium cepa* root meristematic cells exposed to composite wastewater samples collected from different sites and aged tap water during the rainy season are given in Table 6 and during the dry season is given in Table 7. The microscopic appearance of the observed chromosomal abnormalities is given in Figure 3. C-metaphase, chromosomal adherence, bridges, disturbed anaphase, vagrant chromosomes, and chromosomal breaks were the chromosomal abnormalities observed in the present study (Figure 3 and Tables 6 and 7). However, chromosomal breaks were observed only in the samples exposed to the water collected during the dry season (Tables 6 and 7). During both the rainy and dry seasons, significantly high C-metaphase, chromosomal adherence, bridges, and vagrant chromosomes were observed in the *Allium cepa* root meristematic cells exposed to composite wastewater samples collected from the effluent discharge site (site A) compared to the other sites and the control treatment (Tables 6 and 7). In the rainy season, the disturbed anaphase was recorded only from the water samples from site A and the %_0_ occurrence was not significantly different from that of the control treatment (Table 6, ANOVA, Tukey's test, *p* < 0.05). In the dry season, the disturbed anaphase was recorded only in sites B and C and their %_0_ occurrence was not significantly different from that of the control treatment as well (Table 7, ANOVA, Tukey's test, *p* < 0.05).

The occurrence of different types of chromosomal abnormalities indicates the presence of genotoxic agents in the textile effluents [21]. Chromosomal bridges and breaks are categorized as indicators of clastogenic effects which leads to alteration in DNA structure. Chromosomal abnormalities associated with aneugenic effects are chromosome losses, delays, adherence, multipolarity, and C-metaphase [22]. According to Pathiratne et al., the most frequent and easily recognizable chromosomal abnormality in the *Allium cepa* root meristematic cells was vagrant chromosomes [18]. Chromosomal adherence may occur due to the increased chromosomal contraction and condensation [23]. According to the Fiskesjo, presence of chromosomal adherence is considered as a common sign of toxic effects on chromosomes [24]. The unequal distribution of chromosomes or paired chromatids leads to the occurrence of vagrant chromosomes [25]. Stickiness of chromosomes prevent complete separation during anaphase and it may lead to arise of chromosomal bridges [26]. It has been reported that presence of binucleated cells, sticky chromosomes, chromosome fragments, and anaphase bridges in the *Allium cepa* root meristems was induced by the textile effluents [9]. Further, Carita and Marine-Morales reported that there is a high possibility of inducing the chromosomal and nuclear aberrations in *Allium cepa* root meristem cells by textile effluent contaminated with azo dyes and aromatic amines [27, 28]. In the present study, a significantly high number of chromosomal abrasions were observed in the *Allium cepa* root meristematic cells exposed to water collected from the effluent discharge point during both seasons. This indicates the possibility of presence of compounds that induces genotoxic effect in the exposed organisms. Further, the occurrence of these genotoxic and mutation causing compounds may be a result of receiving treated textile effluents over a long period of time and overtime accumulation of trace amounts in the environment.

## 4. Conclusion

The conductivity, TDS, COD, and colour intensity of the water samples collected from the study sites were significantly higher during the dry season compared to those in the rainy season and these changes can be attributed to the dilution effects and increase of water flow rate during the rainy season. The results of the *Allium cepa* bioassay indicate that the mitotic index and phase index in the living cells can be altered and there can be chromosomal abnormalities induced by the treated textile effluents. The occurrence of chromosomal abnormalities indicates the presence of genotoxic agents in the treated textile effluents and there is a possibility that these can cause environmental and health effects in the natural environment if the effluent discharge takes place over a long period of time.

## Figures and Tables

**Table 1:** Spatial variations of physicochemical parameters of wastewater collected from treated textile effluent discharge canal during the rainy season.

Parameter		Site A	Site B	Site C	Site D
pH		8.50 ± 0.06^a^	7.79 ± 0.12^a^	7.80 ± 0.02^a^	6.90 ± 0.01^b^
Temperature (°C)		31.1 ± 0.3^a^	29.4 ± 0.1^b^	29.4 ± 0.1^b^	28.5 ± 0.1^b^
Conductivity (*μ*S/cm)		9263.3 ± 64.4^a^	925 ± 175^b^	916 ± 119^b^	414.00 ± 5.01^c^
TDS (mg/L)		5061.1 ± 32.6^a^	456.4 ± 88.8^b^	450 ± 59.7^b^	199.54 ± 2.31^c^
DO (mg/L)		2.33 ± 0.28^a^	2.82 ± 0.23^a^	2.64 ± 0.14^a^	5.73 ± 0.22^b^
Salinity (‰)		4.49 ± 0.03^a^	0.45 ± 0.09^b^	0.44 ± 0.06^b^	0.20 ± 0.00^c^
BOD_5_ (mg/L)		30 ± 1.2^a^	10 ± 0.6^b^	9 ± 0.2^b^	4 ± 0.1^c^
COD (mg/L)		503 ± 15^a^	60 ± 3^b^	62 ± 12^b^	35 ± 10^b^
Ammoniacal-N (mg/l)		3.1 ± 0.2^a^	2.3 ± 0.1^b^	2.2 ± 0.0^b^	2.1 ± 0.1^b^
Zn		46.5 ± 6.2^a^	38.6 ± 5.3^b^	29.6 ± 4.2^b^	7.5 ± 3.1^c^
Cu		11 ± 2.1^a^	8.9 ± 3.1^b^	7.5 ± 2.1^b^	2.2 ± 0.8^c^
Colour intensities (m^−1^)	436 nm	11.0 ± 0.1^a^	4.2 ± 0.1^b^	4.1 ± 0.4^b^	3.1 ± 0.2^c^
	525 nm	5.5 ± 0.3^a^	3.2 ± 0.0^b^	3.0 ± 0.1^b^	2.4 ± 0.1^c^
	620 nm	3.8 ± 0.4^a^	2.1 ± 0.0^b^	1.8 ± 0.1^b^	1.7 ± 0.1^b^

Data are presented as mean ± standard deviation (SD). Results indicated by different superscript letters in each row are significantly different from each other (*n* = 8, ANOVA, Tukey's test, *p* < 0.05). Site A: effluent discharge point, site B: 100 m downstream from site A, site C: 200 m downstream from site A, and site D: 100 m upstream from site A.

**Table 2:** Spatial variations of physicochemical parameters of wastewater collected from treated textile effluent discharge canal during the dry season.

Parameter		Site A	Site B	Site C	Site D
pH		9.06 ± 0.02^a^	6.96 ± 0.1^b^	6.82 ± 0.07^b^	6.74 ± 0.02^b^
Temperature (°C)		33.1 ± 0.3^a^	31.1 ± 0.2^b^	31.1 ± 0.1^b^	29.7 ± 0.4^b^
Conductivity (*μ*S/cm)		9503 ± 100^a^	4457.8 ± 60^b^	4432.2 ± 45.3^b^	436.67 ± 5.53^c^
TDS (mg/L)		5191.10 ± 62.1^a^	2345.6 ± 30.9^b^	2554.4 ± 98.5^b^	207.58 ± 3.89^c^
DO (mg/L)		2.30 ± 0.21^a^	2.47 ± 0.17^a^	2.59 ± 0.15^a^	5.65 ± 0.09^b^
Salinity (‰)		4.59 ± 0.04^a^	2.16 ± 0.03^b^	2.15 ± 0.02^b^	0.21 ± 0.00^c^
BOD_5_ (mg/L)		36 ± 0.9^a^	12 ± 0.3^b^	10 ± 0.2^b^	6 ± 0.3^c^
COD (mg/L)		673 ± 37^a^	269 ± 6^b^	255 ± 6^b^	176 ± 16^c^
Ammoniacal-N (mg/l)		3.2 ± 0.1^a^	2.9 ± 0.0^ab^	2.3 ± 0.0^b^	2.4 ± 0.0^b^
Zn		42.5 ± 3.5^a^	39.6 ± 6.2^a^	26.6 ± 3.6^b^	8.5 ± 2.1^c^
Cu		10.9 ± 1.1^a^	8.7 ± 1.2^b^	8.5 ± 3.1^b^	2.6 ± 0.5^c^
Colour intensities (m^−1^)	436 nm	16.2 ± 0.1^a^	11.4 ± 0.4^b^	9.5 ± 0.1^c^	8.9 ± 0.1^c^
	525 nm	8.6 ± 0.1^a^	5.8 ± 0.3^b^	4.5 ± 0.1^c^	4.4 ± 0.1^c^
	620 nm	4.5 ± 0.1^a^	2.7 ± 0.2^b^	2.3 ± 0.0^b^	1.9 ± 0.1^c^

Data are presented as mean ± standard deviation (SD). Results indicated by different superscript letters in each row are significantly different from each other (*n* = 8, ANOVA, Tukey's test, *p* < 0.05). Site A: effluent discharge point; site B: 100 m downstream from site A, site C: 200 m downstream from site A, and site D: 100 m upstream from site A.

**Table 3:** Mean mitotic index of root tip cells of *Allium cepa* bulbs following exposure to water collected from study sites during rainy and dry seasons.

Site	Mitotic index (%)	
	Rainy season	Dry season
Site A	**26.4** **±** **1.3**^**a***∗*^	**18.9** **±** **4.1**^**a***∗∗*^
Site B	**29.8** **±** **2.4**^**a***∗*^	**20.9** **±** **3.7**^**a***∗∗*^
Site C	31.5 ± 2.7^b^	29.8 ± 5.0^b^
Site D	48.5 ± 1.8^c^	47.9 ± 4.2^c^
Aged tap water	52.4 ± 5.8^c^	55.6 ± 2.7^c^

Data are represented as mean ± SD. Results indicated by different superscript letters in each column are significantly different from each other. Results indicated by different superscripts each row for site A and site B are significantly different from each other (ANOVA, Tukey's test, *p* < 0.05, *n* = 10).

**Table 4:** The spatial variation of phase index at the cell division phases of *A. cepa* bulbs exposed to composite water samples collected from different sites during the rainy season.

Site	Interphase index (‰)	Prophase index (‰)	Metaphase index (‰)	Anaphase index (‰)	Telophase index (‰)
A	736 ± 32^a^	248 ± 42^a^	5 ± 0.5^a^	5 ± 0.5^a^	3 ± 0.4^a^
B	702 ± 38^a^	288 ± 32^a^	4 ± 0.2^a^	4 ± 0.5^a^	3 ± 0.2^a^
C	685 ± 52^a^	298 ± 32^a^	6 ± 0.1^a^	6 ± 0.5^a^	4 ± 0.2^a^
D	585 ± 45^b^	400 ± 32^a^	6 ± 0.1^a^	2 ± 0.4^a^	8 ± 0.1^b^
Aged tap water	476 ± 15^b^	500 ± 32^b^	5 ± 0.1^a^	6 ± 0.3^a^	6 ± 0.2^b^

Data are presented as mean ± SD. Mean values indicated by different superscript letters at each column are significantly different from each other (ANOVA, Tukey's test, *p* < 0.05, *n* = 10). Site A: effluent discharge point, site B: 100 m downstream from site A, site C: 200 m downstream from site A, and site D: 100 m upstream from site A.

**Table 5:** The spatial variation of phase index at the cell division phases of *A. cepa* bulbs exposed to composite water samples collected from different sites during the dry season.

	Interphase index (%)	Prophase index (%)	Metaphase index (%)	Anaphase index (%)	Telophase index (%)
Site A	781 ± 4^b^ 2^a^	204 ± 32^a^	4 ± 0.4^a^	5 ± 0.24^a^	6 ± 0.54^a^
Site B	750 ± 26^a^	233 ± 25^a^	6 ± 0.5^b^	6 ± 0.24^a^	7 ± 0.54^a^
Site C	702 ± 45^a^	280 ± 24^a^	7 ± 05^b^	7 ± 0.24^a^	7 ± 0.34^a^
Site D	651 ± 23^b^	326 ± 35^b^	10 ± 0.2^c^	7 ± 0.14^a^	7 ± 0.24^a^
Aged tap water	544 ± 56^b^	432 ± 32^b^	11 ± 0.6^c^	6 ± 0.14^a^	8 ± 0.24^a^

Data are presented as mean ± SD. Mean values indicated by different superscript letters at each column are significantly different from each other (ANOVA, Tukey's test, *p* < 0.05, *n* = 10). Site A: effluent discharge point, site B: 100 m downstream from site A, site C: 200 m downstream from site A, and site D: 100 m upstream from site A.

**Table 6:** Chromosomal abnormalities in root meristematic cells exposed to composite wastewater samples collected from different sites and aged tap water in the rainy season.

Site	Chromosomal abnormality (%)					
	C-metaphase	Chromosomal adherence	Bridges	Disturbed anaphase	Vagrant	Chromosomal breaks
Site A	50.0 ± 8.4^a^	66.7 ± 5.6^a^	33.3 ± 5.5^a^	28.0 ± 5.1^a^	100.0 ± 17.6^a^	0.0 ± 0.0
Site B	50.0 ± 5.5^a^	91.7 ± 11.8^a^	0.0 ± 0.0^c^	0.0 ± 0.0^b^	66.7 ± 7.8^b^	0.0 ± 0.0
Site C	33.3 ± 4.8^a^	78.0 ± 9.9^a^	0.0 ± 0.0^c^	0.0 ± 0.0^b^	113.8 ± 16^a^	0.0 ± 0.0
Site D	0.0 ± 0.0^c^	33.3 ± 4.9^b^	0.0 ± 0.0^c^	0.0 ± 0.0^b^	0.0 ± 0.0^c^	0.0 ± 0.0
Aged tap water	18.2 ± 2.0^b^	0.0 ± 0.0^c^	18.2 ± 1.9^b^	29.9 ± 3.8^a^	0.0 ± 0.0^c^	0.0 ± 0.0

Data are presented as mean ± SEM. Means values indicated by different superscript letters at each column are significantly different from each other (ANOVA, Tukey's test, *p* < 0.05). Site A: discharge point, site B: 100 m downstream point, site C: 200 m downstream point, and site D: 100 m upstream point.

**Table 7:** Chromosomal abnormalities in root meristematic cells exposed to composite wastewater samples collected from different sites and aged tap water during the dry season.

Site	Chromosomal abnormality (%)					
	C-metaphase	Chromosomal adherence	Bridges	Disturbed anaphase	Vagrant	Chromosomal breaks
Site A	53.6 ± 7.7^a^	75.0 ± 8.0^a^	73.7 ± 8.9^a^	0.0 ± 0.0^b^	91.9 ± 9.0^a^	25 ± 2.3^a^
Site B	22.2 ± 3.3^b^	62.2 ± 8.0^a^	48.7 ± 6.5^a^	38.9 ± 5.0^a^	95.2 ± 11.1^a^	0.0 ± 0.0^b^
Site C	18.2 ± 1.82^b^	0.0 ± 0.0^c^	28.6 ± 4.8^b^	35.0 ± 4.8^a^	85.2 ± 9.5^a^	22.2 ± 3.2^a^
Site D	0.0 ± 0.0^c^	0.0 ± 0.0^c^	0.0 ± 0.0^c^	0.0 ± 0.0^b^	79.5 ± 9.0^a^	16.7 ± 2.4^a^
Aged tap water	0.0 ± 0.0^c^	20.0 ± 3.2^b^	0.0 ± 0.0^c^	34.6 ± 2.8^a^	28.6 ± 4.5^b^	0.0 ± 0.0^b^

Data are presented as mean ± SEM. Means values indicated by different superscript letters at each column are significantly different from each other (ANOVA, Tukey's test, *p* < 0.05). Site A: discharge point, site B: 100 m downstream point, site C: 200 m downstream point, and site D: 100 m upstream point.

## Data Availability

The toxicology and water quality data used to support the findings of this study are available from the corresponding author upon request.
